# Risk Factors for Graft Failure and Death following Geriatric Renal Transplantation

**DOI:** 10.1371/journal.pone.0153410

**Published:** 2016-04-13

**Authors:** Hyungjin Cho, Hoon Yu, Eunhye Shin, Young Hoon Kim, Su-Kil Park, Min-Woo Jo

**Affiliations:** 1 Division of Nephrology, Department of Internal Medicine, University of Ulsan College of Medicine, Asan Medical Center, Seoul, Korea; 2 Department of Internal Medicine, National Rehabilitation Center, Seoul, Korea; 3 Department of Surgery, Asan Medical Center and University of Ulsan College of Medicine, Seoul, Korea; 4 Department of Preventive Medicine, University of Ulsan College of Medicine, Seoul, Korea; University of Toledo, UNITED STATES

## Abstract

**Background:**

Population aging is a major health concern in Asian countries and it has affected the age distribution of patients with end-stage renal disease (ESRD). As a consequence, the need for kidney transplantation in the geriatric population has increased, but the shortage of donors is an obstacle for geriatric renal transplantation. The aim of this study was to evaluate risk factors for graft failure and death in geriatric renal transplantation.

**Methods:**

Kidney transplantations performed in a tertiary hospital in South Korea from May 1995 to December 2014 were retrospectively reviewed. Recipients younger than 60 years of age or who underwent other organ transplantations were excluded. The Kaplan-Meier method was used to assess patient and graft survival. A Cox regression analysis was used to evaluate risk factors for graft failure and patient death.

**Results:**

A total of 229 kidney transplantation patients were included. Graft survival at 1, 5, and 10 years were 93.2%, 82.9%, and 61.2% respectively. Patient survival at 1, 5, and 10 years were 94.6%, 86.9%, and 68.8%, respectively. According to the Cox multivariate analysis, ABO incompatibility (hazard ratio [HR] 3.91, p < 0.002), DGF (HR 3.544, p < 0.004), CMV infection (HR 2.244, p < 0.011), and HBV infection (HR 6.349, p < 0.015) were independent risk factors for graft survival. Recipient age (HR 1.128, p < 0.024), ABO incompatibility (HR 3.014, p < 0.025), CMV infection (HR 2.532, p < 0.010), and the number of HLA mismatches (HR 1.425, p < 0.007) were independent risk factors for patient death.

**Conclusion:**

Kidney transplantation in the geriatric population showed good clinical outcomes. ABO incompatibility, DGF, CMV infection, and HBV infection were risk factors for graft failure and the recipient age, ABO incompatibility, CMV infection, and the number of HLA mismatches were risk factors for patient death in geriatric renal transplantation.

## Introduction

Kidney transplantation (KT) is known to have several benefits compared to other renal replacement therapies, such as hemodialysis (HD) or peritoneal dialysis (PD). KT increases life expectancy of patients with chronic kidney disease who require renal replacement therapy [1,2], and it brings quality of life improvement even if the recipients are elderly [3,4].

Aging has progressed rapidly in Asian countries [5], and it took less than 30 years for many Asian countries to shift from an aging society to an aged society[6]. This phenomenon has affected the age distribution of patients with end-stage renal disease (ESRD). According to the Japanese Society for Dialysis Therapy, 14% of Japanese patients who underwent dialysis therapy were aged 80 years in 2004, and this proportion increased to 22% in 2012 [7]. Likewise, 19% of Korean patients who started dialysis for renal replacement therapy were >65 years old in 2000, but this rate increased to 37.5% in 2012 [8].

Aging of ESRD patients has led to an increased requirement for KT in the geriatric population. According to the Korean Network for Organ Sharing (KONOS), only 0.02% of KT recipients in 2000 were >60 years old; whereas, in 2014 11.17% of KT recipients were >60 years old [8].

Although KT has several benefits compared to other renal replacement therapies, KT accounts for only 20% of all renal replacement therapy. Donor shortage is most important cause of this situation, which is a more critical problem in geriatric individuals than in younger patients. Moreover, the deceased donor kidney allografts tend to be allocated to younger recipients when patients are in similar conditions. Also, living donor candidates for geriatric recipients tend to be old, and many of them have limitations for organ donation because of their health problems. Therefore, evaluating the risk factors of graft failure and patient death after KT is important, especially in the geriatric population. However, there are few data available regarding these risk factors, especially in Asian populations [9]. The aim of this study was to evaluate the risk factors for graft failure and patient death in geriatric renal transplantation.

## Materials and Methods

### Study population

Patients who received KTs from May 1995 to December 2014 at Asan Medical Center, a tertiary hospital in South Korea, were included. Recipients younger than 60 years and who received transplantation of other organs, such as the liver, pancreas, or heart, were excluded. Medical records of the included patients were retrospectively reviewed. All research procedures were approved by the Institutional Review Board at Asan Medical Center (2015–0590). All information about patients were anonymized prior to analysis. None of the transplant donors were from a vulnerable population and all donors or next of kin provided written informed consent that organ donation was pro bono.

### Outcome variables

We gathered information on the age of recipients and donors and sex of the recipients as demographic data. Information on the dialysis vintage, mode of dialysis, causes of ESRD, number of human leukocyte antigen (HLA) mismatches, ABO incompatibility, pre-transplant co-morbidities (diabetes mellitus, hypertension, coronary artery disease, stroke, and chronic hepatitis B [HBV] and C [HCV]), immunosuppressant agent used, previous transplantation history, cold ischemic time, donor type, and body mass index (BMI) were collected as independent variables. Hypertension was defined as a systolic blood pressure≥140mmHg or a diastolic blood pressure of ≥90mmHg or those who were receiving antihypertensive medication at the time of transplantation. Prior to transplantation, all recipients were evaluated their cardiac function with transthoracic echocardiography and thallium-201 myocardial perfusion scintigraphy. If abnormal findings were revealed by those studies, recipients were referred to a cardiologist. Coronary artery disease was defined as a previous history of percutaneous coronary intervention or coronary artery bypass graft surgery.

Information on adverse events (delayed graft function [DGF], cytomegalovirus [CMV] infection, BK virus [BKV] infection, and biopsy proven rejection) after transplantation was also collected. DGF was defined as a requirement for dialysis within a week after transplantation. CMV infection was defined as the presence of CMV antigenemia or clinical symptoms of specific organ infection accompanied with a positive result on culture, histopathology, immunohistochemical analysis, or *in situ* hybridization from a sample of a specific organ [10]. Two hundred nine patients (92.9%) showed positive CMV immunoglobulin G prior to transplantation. One hundred ninety donors showed positive CMV immunoglobulin G and 6 donors showed negative results prior to transplantation. Rest of the recipients and donors were not checked CMV IgG prior to transplantation. According to the Kidney Disease Improving Global Outcome (KDIGO) guidelines [11], a plasma BKV level of >10,000 copies/mL is associated with a 93% specificity for the presence of BKV nephropathy. Therefore, in our present study, BKV infection was defined as a plasma BKV level of >10,000 copies/mL.

Graft failure and patient death were collected as outcome variables. Graft failure was defined as re-starting any type of renal replacement therapy or death of the recipient. Acute renal dysfunction that occurred with septic shock, bleeding, and any other causes was not considered as graft failure. However, renal dysfunction that required renal replacement therapy for more than 3 months was considered as graft failure. Information on the cause of death was also collected.

### Statistical analysis

The Kaplan-Meier method was used to assess patient survival and graft survival. Cox regression analysis was used to evaluate the potential risk factors for graft failure and patient death, in which we used 10 variables in the order of p value obtained from the univariate analysis to avoid overfitting. Fisher’s exact test was used to evaluate correlation between ABO incompatible KT and death by infectious complication. A P-value of ≤0.05 was used to represent statistical significance. SPSS software version 20.0 was used for the statistical analyses (IBM Corp., Armonk, NY).

## Results

During the study period, 229 KTs were performed for recipients aged over 60 years. Four patients who received transplantation of other organs were excluded. Ultimately, a total of 225 patients were included (See S1 Dataset). The mean follow-up period was 57.9 ±49.6 months. Table 1 presents the baseline characteristics of the study patients. The mean age of the recipients was 63.2 ± 2.7 years, and 63.6% were male. The most common cause of ESRD was diabetes (38.2%, n = 86) and hypertension was the second common cause (14.2%, n = 32). The mean time on dialysis prior to transplantation was 30.6 ± 45.0 months. At the time of transplantation, 89.3% of recipients (n = 201) had hypertension, 41.3% (n = 93) had diabetes, 18.2% (n = 41) had history of coronary artery disease, 10.2% (n = 23) had stroke, 2.2% (n = 5) had chronic hepatitis B and one recipient had chronic hepatitis C. Living donor transplantation accounted for 79.6% of all transplantations. The mean age of donors was 43.1 ± 12.7 years. At baseline, 44.4% of recipients took cyclosporine-based immune suppressive therapy, while 52.4% took tacrolimus-based immune suppressive therapy. CMV infection occurred in 20.4% of recipients (n = 46)and 9.3% experienced BKV infection (n = 21). Biopsy proven rejections were reported in 16.9% of all patients (n = 38).

**Table 1:** Baseline characteristics of the study subjects.

**Recipient**			
	Demographics		
		Age (mean ± SD)	63.2 ± 2.7
		Male, n (%)	143 (63.6)
		BMI (mean ± SD)	23.4 ± 2.7
	Cause of ESRD, n (%)		
		Glomerulonephritis	25 (11.1)
		DM	86 (38.2)
		HTN	32 (14.2)
		PCKD	8 (3.6)
		Others	16 (7.1)
		Unknown	58 (25.8)
	Mode of dialysis, n (%)		
		Pre-emptive KT	39 (17.3)
		HD	156 (69.3)
		PD	22 (9.8)
		HD+PD	8 (3.6)
	Dialysis vintage, months (mean ± SD)		30.6 ± 45.0
	Co-morbidity		
		DM, n (%)	93 (41.3)
		HTN, n (%)	201 (89.3)
		CAD, n (%)	41 (18.2)
		Stroke, n (%)	23 (10.2)
		HBV, n (%)	5 (2.2)
		HCV, n (%)	1 (0.4)
**Donor**			
	Living donor, n (%)		179 (79.6)
	Donor age (mean ± SD)		43.1 ±12.7
**Transplantation characteristics**			
	ABO incompatible, n (%)		31 (13.8)
	Re-transplantation, n (%)		16 (7.1)
	Cold ischemic time, min (mean ± SD)		85.8 ±117.9
Immunosuppressant			
	Calcineurin inhibitor, n (%)		
		Cyclosporine	100 (44.4)
		Tacrolimus	118 (52.4)
	Antimetabolite, n (%)		
		Azathioprine	24 (10.7)
		Mycophenolate mofetil	161 (71.6)
**Adverse event**			
	DGF, n (%)		18 (8.0)
	CMV, n (%)		46 (20.4)
	BKV, n (%)		21 (9.3)
	Biopsy proven rejection		38 (16.9)
	Graft failure		52 (23.1)
	Death		40 (17.8)

BMI, body mass index; ESRD, end-stage renal disease; DM, diabetes mellitus; HTN, hypertension; PCKD,; polycystic kidney disease; KT, kidney transplantation; HD, hemodialysis; PD, peritoneal dialysis; CAD, coronary artery disease; HBV, hepatitis B virus; HCV, hepatitis C virus; DGF, delayed graft failure; CMV, cytomegalovirus; BKV, BK virus

**Fig 1:**
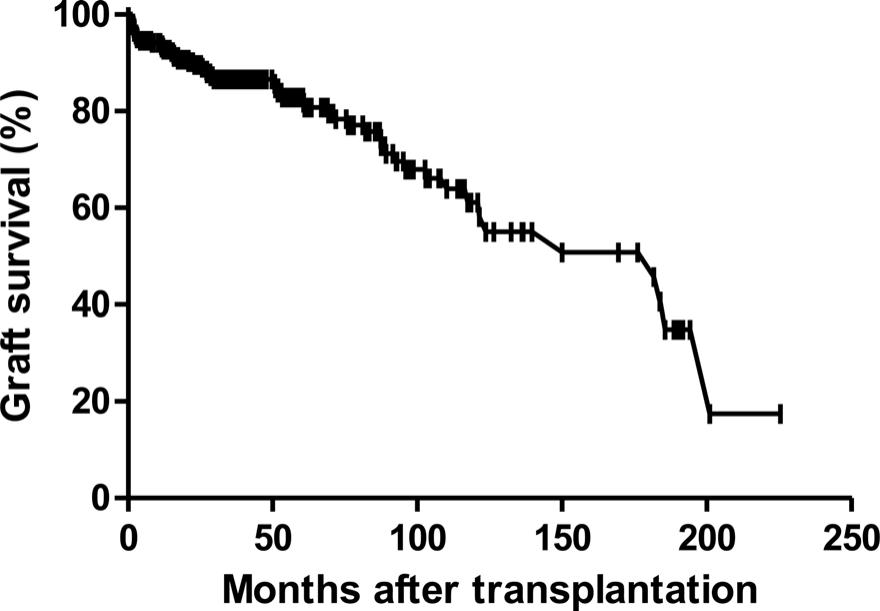
Graft survival rates following kidney transplantation in the Korean geriatric recipients.

**Fig 2:**
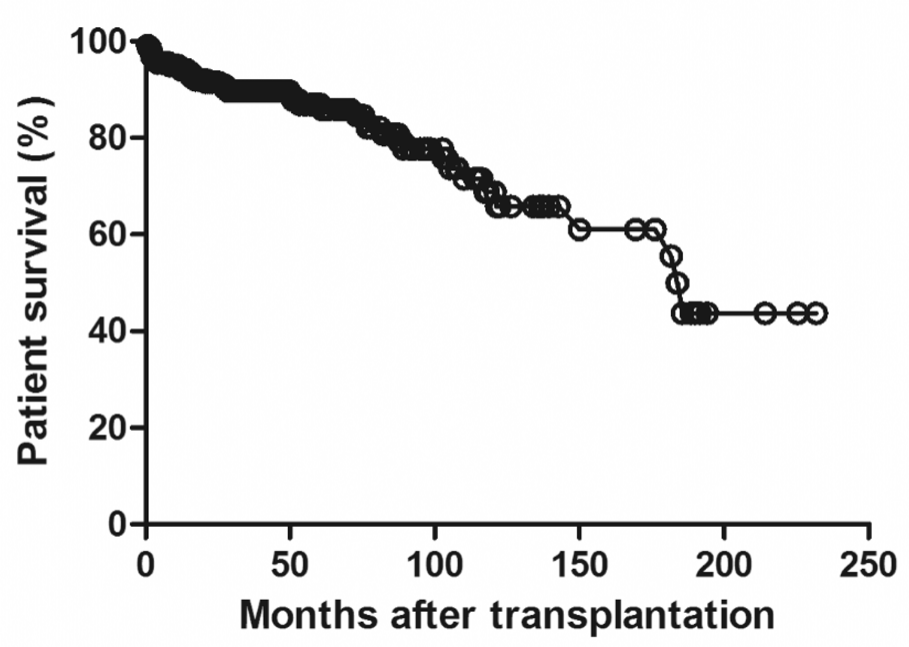
Patient survival rates following kidney transplantation in the Korean geriatric recipients.

Fig 1 shows the graft survival in the geriatric recipients. Graft survival at 1, 5, and 10 years were 93.2%, 82.9% and 61.2%, respectively. Fig 2 shows the patient survival and patient survival at 1, 5, and 10 years were 94.6%, 86.9% and 68.8%, respectively. During the study period, 52 cases of graft failure occurred. The most common cause of graft failure was patient death (76.9%, n = 40). Univariate analysis revealed that the dialysis time prior to transplantation, ABO incompatibility, DGF, and CMV infection were risk factors for graft failure. According to the Cox multivariate analysis, ABO incompatibility (Hazard ratio [HR] 3.91, p < 0.002), DGF (HR 3.544, p < 0.004), CMV infection (HR 2.244, p < 0.011), and HBV infection (HR 6.349, p < 0.015) were independent risk factors. (Table 2)

**Table 2:** Risk factors for graft failure.

	Univariate analysis			Multivariate analysis		
	HR	95% CI	P value	HR	95% CI	P value
Recipient age	1.076	0.979–1.184	0.129			
Recipient sex	1.197	0.687–2.088	0.525			
Donor age	1.011	0.989–1.034	0.322			
Living donor	0.694	0.375–1.285	0.245			
Time on dialysis prior to transplantation (per month)	1.005	1.000–1.011	0.049			
No. of HLA mismatches	1.227	0.986–1.527	0.067	1.243	0.995–1.552	0.055
ABO incompatible	2.875	1.225–6.751	0.015	3.910	1.676–9.123	0.002
Delayed graft function	3.752	1.633–8.619	0.002	3.544	1.511–8.311	0.004
CMV	2.610	1.436–4.745	0.002	2.244	1.205–4.177	0.011
HBV	3.503	0.833–14.731	0.087	6.349	1.434–28.114	0.015
HCV	3.964	0.540–29.121	0.176			

CMV, cytomegalovirus; HBV, hepatitis B virus; HCV, hepatitis C virus.

Forty patients died during the follow-up period. More than half of these deaths were caused by infection (52.5%, n = 21), and this was the most common cause of death (Table 3). Cardiovascular disease was followed by infection. Univariate analysis showed that time on dialysis prior to transplantation, the number of HLA mismatches, ABO incompatibility, and CMV infection were risk factors for patient death. Cox multivariate analysis showed that recipient age (HR 1.128, p < 0.024), ABO incompatibility (HR 3.014, p < 0.025), CMV infection (HR 2.532, p < 0.010), and the number of HLA mismatches (HR 1.425, p < 0.007) were independent risk factors for patient death. (Table 4)

**Table 3:** Cause of death in the study population according to the type of donor.

Type of donor		Cause of death	No. of patients
**Deceased donor (n = 46)**			
		Infection	9
		Cardiovascular disease	1
		Unknown	4
**Living donor (n = 179)**			
	ABO compatible (n = 148)		
		Infection	8
		Malignancy	2
		Cardiovascular disease	3
		Unknown	7
	ABO incompatible (n = 31)		
		Infection	4
		Bleeding	1
		Unknown	4

**Table 4:** Risk factors for patient death.

	Univariate analysis			Multivariate analysis		
	HR	95% CI	P value	HR	95% CI	P value
Recipient age	1.107	0.997–1.229	0.056	1.128	1.016–1.253	0.024
Recipient sex	0.950	0.496–1.821	0.877			
Donor age	1.015	0.989–1.041	0.268			
Donor type	0.518	0.267–1.005	0.052			
Time on dialysis prior to transplantation (per month)	1.006	1.000–1.012	0.040	1.006	1.000–1.012	0.054
No. of HLA mismatches	1.373	1.067–1.768	0.014	1.425	1.103–1.840	0.007
ABO incompatibility	3.124	1.221–7.993	0.018	3.014	1.145–7.930	0.025
CMV	2.712	1.388–5.297	0.004	2.532	1.254–5.112	0.010
BKV	0.220	0.030–1.599	0.134	0.238	0.032–1.759	0.160
HCV	5.742	0.773–42.639	0.088			

CMV, cytomegalovirus; BKV, BK virus; HCV, hepatitis C virus.

In order to evaluate if infection, as the leading cause of death in our study subjects, differed between in ABO compatible and incompatible KT, we compared the ratio of death caused by infection between the two groups. ABO incompatible KT was introduced since November 2009 in our center, therefore, we evaluated data of patients who underwent living donor transplantation after this time point. A total of 110 patients were subject to this analysis, and as a result, death caused by infection was significantly higher in ABO incompatible group (13.8%, n = 4) than in ABO compatible group (2.5%, n = 2, p = 0.041).

## Discussion

In this study, renal transplantation in the elderly showed fairly good transplantation outcomes. Our investigation also revealed that ABO incompatibility, DGF, CMV infection, and HBV infection were risk factors for graft failure and that the recipient age, ABO incompatibility, CMV infection, and the number of HLA mismatches were risk factors for patient death in geriatric renal transplantation. Our findings were similar to those from previous studies, as DGF [12] and CMV infection [13,14] were proven to be risk factors for graft failure, and recipient age [15] and CMV infection [16,17] were independent risk factors for patient survival.

Baseline characteristics showed that mean donor age was significantly younger than recipient age, because most allograft were donated from living donor. In Korea, living donor KT was more commonly performed than deceased donor KT compared to other western countries and this trend was reflected in our study. According to KONOS 2014 annual report, in 2013, the deceased donor kidney transplant rate was 15.22 per million population in Korea, while it was 38.58 per million population in the United States. On the contrary, in the very same period, the living donor kidney transplant rate was 20.48 per million population in Korea, while it was 18.03 per million population in the United States [8].

Contrary to previous studies [18,19], ABO incompatible KT was shown as one of the independent risk factors for patient death and graft failure in our study. Prior to ABO incompatible KT, recipients were administered 200 mg of rituximab, and 3 to 7 sessions of plasmapheresis were performed 3 to 14 days before transplantation in our center. There were two exceptional cases of receiving 500mg of rituximab since they had underwent transplantation before November 2009. The desensitization protocol was applied identically for both elderly and younger recipients. The elderly patients are not only vulnerable to infectious complications [20–22], but the desensitization procedure could have added risk to infection, coming from the higher immunosuppression in the process of ABO incompatible KT. In fact, our results showed higher percentage of death caused by infection in patients who received ABO incompatible KT compared with those who received ABO compatible KT. Several studies accomplished in western countries revealed that the most common cause of patient death in geriatric KT was a cardiac problem [23,24]. By contrast, studies carried out in Asian countries, including the present study, indicate that the most common cause of patient death to be infection [9]. A reduced dose ABO incompatible KT protocol for elderly recipients may improve KT outcomes. In this regard, Uchida et al. reported a successful clinical outcome of ABO incompatible KT with a reduced dose desensitization protocol in a geriatric group [25].

CMV infection is a risk factor for graft failure and patient death. In this study, we used a preemptive strategy against CMV infection. However, Lai et al. reported that even though recipients received low-dose ganciclovir (1.5 g/day for 3 months) prophylaxis, the therapy could be a risk factor for patient death [9]. For elderly recipients, an aggressive CMV infection prevention strategy could improve clinical outcomes of KT.

Previously, HBV infection was reported to have an adverse effect on graft survival in renal transplantation, but recent studies have shown no difference in the graft survival between HBsAg positive and negative recipients. Development of new antiviral agents, such as entecavir or tenofovir, is attributed to this change. Huang et al. reported that entecavir was superior to lamivudine in preventing reactivation in lymphoma patients who underwent chemotherapy [26]. In our present study, HBV infection was revealed to be a risk factor for graft failure. Five HBsAg positive recipients were included in our current series. Of these patients, only one patient experienced graft loss and HBV reactivation. This patient had received lamivudine after KT, but lamivudine resistance had occurred. Two other recipients were administered entecavir, and the remaining patients received tenofovir. All four patients still have a functioning graft and have not experienced HBV reactivation. HBsAg-positive geriatric patients who are taking new antiviral agents, such as entecavir or tenofovir are expected to undergo renal transplantation safely.

This study had several limitations. Firstly, the number of subjects in the study population was relatively small. This could be due to the fact that geriatric renal transplantation is not commonly performed in Korea. In fact, only 9.1% of total renal transplantations (8,283 renal transplantations from 2010 to 2014) were performed in patients over 60 years old [8]. In order to overcome this limitation, we tried to extend the study period as long as we could and used 60 years as the cut off age for defining geriatric population. Secondly, there could be a selection bias due to the nature of single center observational study. Especially, the proportion of deceased donor was relatively small because old patients have low priority for KT in the KONOS and deceased donor KT rate was actually reported lower than living donor KT rate in Korea [8]. However, this reflects the current status of KT in Korea. Based on our findings, as the volume of elderly patients who receive KT increases, these issues are expected to be solved. In addition, further multi-center studies should be conducted.

## Conclusion

The clinical outcomes of geriatric renal transplantation were good. We found that ABO incompatibility, DGF, CMV infection, and HBV infection were risk factors for graft failure and the recipient age, ABO incompatibility, CMV infection, and the number of HLA mismatches were risk factors for patient death in geriatric renal transplantation. In order to improve transplantation outcomes in geriatric population, we need to develop new modified desensitization protocol for ABO incompatible KT recipients, more aggressive CMV prophylaxis strategy and we should actively use newly developed antiviral agents such as entecavir or tenofovir in HBsAg positive recipients. However, all of these factors should be confirmed by further prospective, randomized and multicenter studies.

## Supporting Information

S1 Dataset Baseline characteristics of the study population. (XLSX)
